# Quantitative Computed Tomographic Descriptors Associate Tumor Shape Complexity and Intratumor Heterogeneity with Prognosis in Lung Adenocarcinoma

**DOI:** 10.1371/journal.pone.0118261

**Published:** 2015-03-04

**Authors:** Olya Grove, Anders E. Berglund, Matthew B. Schabath, Hugo J. W. L. Aerts, Andre Dekker, Hua Wang, Emmanuel Rios Velazquez, Philippe Lambin, Yuhua Gu, Yoganand Balagurunathan, Edward Eikman, Robert A. Gatenby, Steven Eschrich, Robert J. Gillies

**Affiliations:** 1 Department of Cancer Imaging and Metabolism, H. Lee Moffitt Cancer Center and Research Institute, Tampa, FL, United States of America; 2 Department of Biomedical Informatics, H. Lee Moffitt Cancer Center and Research Institute, Tampa, FL, United States of America; 3 Department of Cancer Epidemiology, H. Lee Moffitt Cancer Center and Research Institute, Tampa, FL, United States of America; 4 Department of Radiology, H. Lee Moffitt Cancer Center and Research Institute, Tampa, FL, United States of America; 5 Department of Radiation Oncology, MAASTRO clinic, Research Institute GROW, Maastricht University, 6229ET Maastricht, The Netherlands; 6 Department of Radiology, Tianjin Medical University Cancer Institute and Hospital, National Clinical Research Center of Cancer, Key Laboratory of Cancer Prevention and Therapy, Tianjin, China; 7 Department of Radiation Oncology, Dana-Farber Cancer Institute, Brigham and Women’s Hospital, Harvard Medical School, Boston, MA, United States of America; 8 Department of Radiology, Brigham and Women’s Hospital, Harvard Medical School, Boston, MA, United States of America; Universidad Carlos III of Madrid, SPAIN

## Abstract

Two CT features were developed to quantitatively describe lung adenocarcinomas by scoring tumor shape complexity (feature 1: convexity) and intratumor density variation (feature 2: entropy ratio) in routinely obtained diagnostic CT scans. The developed quantitative features were analyzed in two independent cohorts (cohort 1: n = 61; cohort 2: n = 47) of patients diagnosed with primary lung adenocarcinoma, retrospectively curated to include imaging and clinical data. Preoperative chest CTs were segmented semi-automatically. Segmented tumor regions were further subdivided into core and boundary sub-regions, to quantify intensity variations across the tumor. Reproducibility of the features was evaluated in an independent test-retest dataset of 32 patients. The proposed metrics showed high degree of reproducibility in a repeated experiment (concordance, CCC≥0.897; dynamic range, DR≥0.92). Association with overall survival was evaluated by Cox proportional hazard regression, Kaplan-Meier survival curves, and the log-rank test. Both features were associated with overall survival (convexity: p = 0.008; entropy ratio: p = 0.04) in Cohort 1 but not in Cohort 2 (convexity: p = 0.7; entropy ratio: p = 0.8). In both cohorts, these features were found to be descriptive and demonstrated the link between imaging characteristics and patient survival in lung adenocarcinoma.

## Introduction

Lung cancer is the leading cause of cancer death in the U.S. and worldwide[1]. Despite therapeutic advances, the overall 5-year survival remains disappointingly low, at around 16%. Clinical decisions for the treatment of lung cancer are largely based on patient characteristics such as performance status, stage at diagnosis, and tumor histology. However, the clinical and biological heterogeneity within histological subtypes remain a major roadblock to successfully treatment of the disease as histologically similar tumors display a wide range of treatment response and metastatic behavior[2].

More recently, treatment strategies have begun to involve the subdivision of non-small-cell lung cancers (NSCLC) into molecular subsets based on specific driver mutational status in oncogenes and tumor suppressor genes [3,4]. Recent works have demonstrated a link between imaging features and gene expression patterns[5–8] thus highlighting the potential of imaging features to be used as independent prognostic or predictive biomarkers essential for enhancing the clinical decision making process. It is expected that the changes at the molecular level will be observable as related imaging phenomena[9]. Tumors within the same histological subtype demonstrate variable and definable imaging characteristics [10]. We propose that these characteristics can be quantified and used in addition to clinical and molecular characteristics to enhance medical decision-making process.

While complete genome profiling has not yet been adapted into the clinic, radiographic imaging is routinely performed on most patients. Computer tomography (CT) has remained an important diagnostic tool used for initial tumor assessment and staging in lung cancer. CT imaging interrogates the entire tumor ‘in situ’ in the context of its environment and can thus be used to assess the tumor globally. Additionally, it can be used to describe tumor heterogeneity and sub-regional “habitats” within the tumor[11]. Due to the increasing number of therapy options for NSCLC patients, these patient-specific prognostic biomarkers have the potential of individualizing and thus improving patient care and outcome.

NSCLC tumors are routinely characterized, using diagnostic imaging, based on their size, shape and margin morphology, and the extent of internal enhancement and necrosis. However, the terminology used in radiology to characterize the pathological findings remains subjective and the underlying data are rarely quantified; hence, we contend that their full potential to support medical decision making is underutilized. Quantifying these observations with computer assistance could provide systematic prognostic information with minimal inter- and intra-reader variability. Furthermore, quantitative data can be stored in databases, allowing these data to be mined to develop models for improved diagnosis, prognosis and prediction [12].

Although there has been an increase in research activity in the areas of lung nodule detection and classification using image processing and data mining algorithms, few investigators have pursued the development of diagnostic CT-based prognostic imaging biomarkers in NSCLC[9,13].In this work, we quantitatively analyzed diagnostic CT scans of lung adenocarcinomas to develop prognostic imaging features. In order to minimize genetic heterogeneity, we focused our research on lung adenocarcinomas, the most common histological subtype of lung cancer [14]. Diagnostic scans were collected retrospectively and augmented with patient information and clinical follow-up data, which enabled us to develop and test models to predict survival. Since the data were collected retrospectively as part of routine clinical practice, there was variability in terms of instrumentation, image acquisition and reconstruction parameters. We therefore recognized the importance of developing imaging features that were robust across the wide variability encountered in clinically-acquired diagnostic scans.

The development of the proposed imaging features was driven by the hypothesis that tumor shape and intratumor density variation reflect tumor biology and systematic quantification of these imaging characteristics can be used to describe tumor development and patient survival. Both features consistently scored tumors according to the pursued characteristics and showed prognostic behavior. Furthermore, the features were tested for reproducibility under standard patient related variations which showed high degree of reproducibility.

## Materials and Methods

### Ethics Statement

The University of South Florida institutional review board approved this retrospective study and waived the informed consent requirement. Data were collected and handled in accordance with the Health Insurance Portability and Accountability Act. Patient data was anonymized and de-identified prior to the analyses.

### Study population and data

The protocol for this retrospective study, including the participation of the MAASTRO clinic, was approved by the Institutional Review Board (IRB). Imaging and clinical data were obtained on patients diagnosed with primary lung adenocarcinoma who were treated in the Thoracic Oncology Program at the H. Lee Moffitt Cancer Center and Research Institute and the Maastricht Radiation Oncology Clinic (MAASTRO). The Moffitt cohort (Cohort 1) included 61 patients and orthogonal MAASTRO cohort (Cohort 2) included 47 patients. Inclusion criteria encompassed patients who underwent surgical resection and had corresponding pre-surgery diagnostic CTs obtained within 60 days of the diagnosis.

For each patient, the cohorts included de-identified diagnostic pre-treatment contrast-enhanced CT scans acquired between years 2006 and 2009 as well as clinical data including demographics, diagnosis, TNM stage, and patient survival.

Clinical data were obtained from Moffitt’s cancer registry, which abstracts self-report information and clinical data from patient medical records including demographics, diagnosis, stage, and treatment. Follow-up for vital status information occurs annually through passive and active methods. Pathologic TNM staging was utilized when available and clinical stage was used if these pathologic staging was missing. For this analysis smoking status was categorized as self-report ever smoker (current or former smoker) or never smoker.

### Tumor and lung segmentation

Patient CT scans were segmented to identify lung fields and tumors. The delineation of the lung fields was carried out using single click ensemble algorithm developed using Lung Tumor Analysis (LuTA) tool within the Definiens Developer XD© (Munich, Germany) software platform. Target lesions were volumetrically segmented using semiautomatic approach. The resident radiologist (over 2 years of experience) oversaw the segmentation boundaries on the CT slices. Performing semi-automatic segmentation not only decreased user interaction and eliminated the need for a manually drawn boundary, but also provided robust, reproducible and consistent delineation of the tumor region across the CT slices. We have previously demonstrated that the single click ensemble segmentation algorithm reduced inter-observer variability while capturing the intricacies and important details of the tumor boundary[15].

Algorithms for image feature extraction and quantification of the segmented tumor regions were implemented in MATLAB (Mathworks, Natick, MA).

### Convexity morphological feature

Convexity algorithm (S1 Fig.) was developed to quantify shape variation of the tumor border. Irregularities along tumor perimeter can result from intratumor heterogeneity and differences in growth patterns, interaction with the surrounding environment and spiculations, multiple finger-like projections into the parenchyma which are generally considered to be a poor prognostic indicator[14,16].

**Fig 1:**
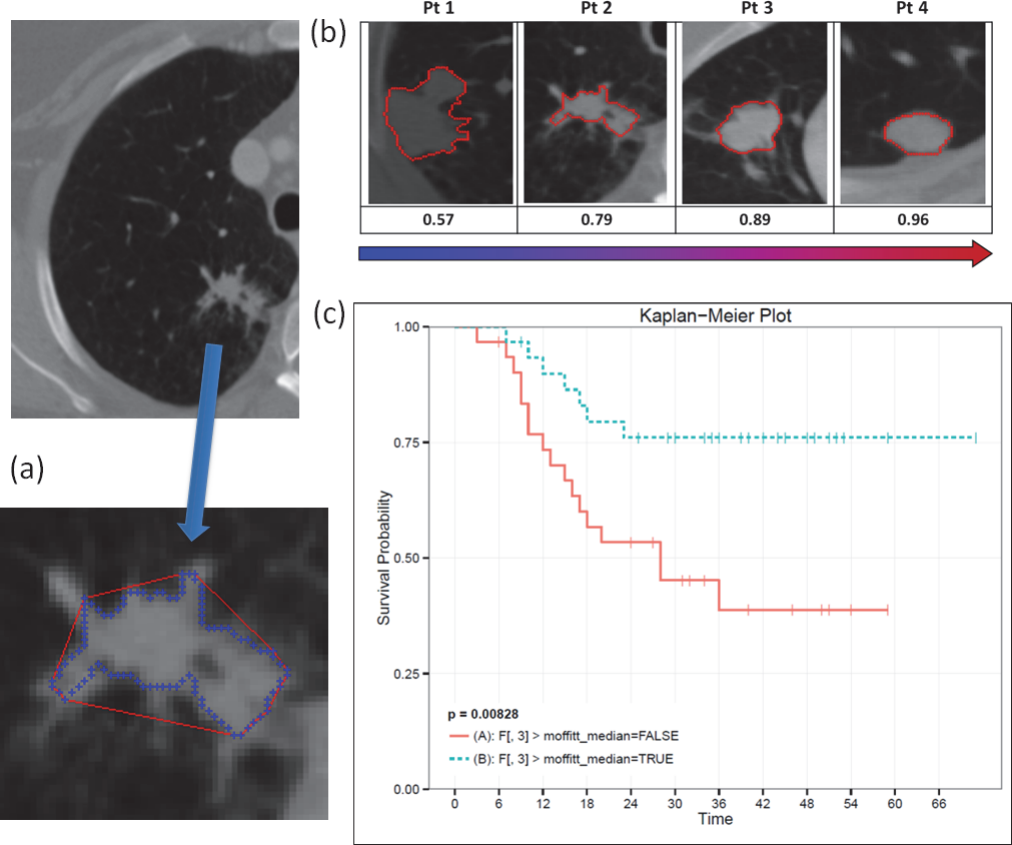
Convexity feature was developed to quantify tumor shape. Convexity is computed as a ratio of tumor border (blue) to convex hull (red) (a). Convexity feature tracks the change in tumor morphology (b). Convexity is predictive of patient overall survival when dichotomized at the median value (c).

The convexity of the tumor border was calculated as the ratio of the areas contained within a) the tumor perimeter to b) the calculated convex hull (Fig. 1A). The convex hull was computed by defining the smallest convex polygon enclosing a planar tumor region of interest (ROI) using the implementation of QuickHull algorithm[17]. Using tumor segmentation mask for a given CT slice as input, the divide and conquer approach automatically computed a convex hull vector of pixel locations. Intrinsically the ratio between the area of the tumor mask and its convex hull described the amount of substantial protuberances and depressions along the tumor border.

In order to account for all sequential slices containing tumor ROI, the calculation was performed individually on each slice and a mean score was computed using all slices for the tumor. A convexity score of *one* corresponded to a shape that does not present with any concavities along its perimeter. Convexity scores less than one measure the degree of deviation from a convex shape.

Since tumor pressing against the pleural wall can compromise the appearance of tumor morphology, automatic pleural attachment detection was incorporated into the convexity feature algorithm. For each CT slice, perimeters from segmented tumor and lung regions were extracted (S2 Fig.). Pleural attachment was determined if perimeters overlapped. If for a particular slice more than half of the tumor perimeter pixels were also in the lung perimeter, its convexity score was eliminated from the mean score calculation. This step allowed us to account for significant cases of shape smoothing that resulted from tumor pressing against the pleural wall.

### Entropy Ratio of intratumor intensity variation feature

Entropy ratio feature (S1 Fig.) was developed to score heterogeneity of pixel attenuation coefficients across the tumor. The entropy filter is the implementation of Shannon entropy. In the image processing context, entropy is the measure of variation computed on the pixel histogram distribution within a given ROI. It is defined as $\sum_{i\;=\;1}^{n\;=\;255}-p_{i}{log}_{2}p_{i}$ where *p*_i_ represents the probability (normalized frequency) of the given intensity value *i*. In our study we used a 256 intensity bins.

Segmented tumor mask was subdivided into two distinct regions: core and boundary. The subdivision was driven by the hypothesis that these distinct regions reflect unique, spatially explicit biological processes, e.g. necrosis in the core and proliferation along the periphery, and should therefore be assessed separately. Tumor growth and interaction with the surrounding microenvironment has been shown to lead to intratumor changes observable in radiographic scans[5]. In addition, the introduced spatial constraint helped account for edge effect manifesting itself in higher changes in intensities at the tumor interface which would otherwise skew the calculations had the summary statistics across the entire tumor ROI been applied.

Core and boundary masks were generated automatically from the original tumor segmentation using a series of morphological operations, erosion and dilation (S3 Fig.). Two disk-shaped structural elements with radii of 3 or 5 pixels were used. A binary segmentation mask for each tumor slice was first dilated using a disk with a 3 pixel radius. This produced a dilated mask which was then eroded using a disk with a 5 pixel radius to generate the core region mask. The ‘doughnut shape’ mask for the boundary region was produced by subtracting the core mask from the dilated mask (S3 Fig.).

**Fig 2:**
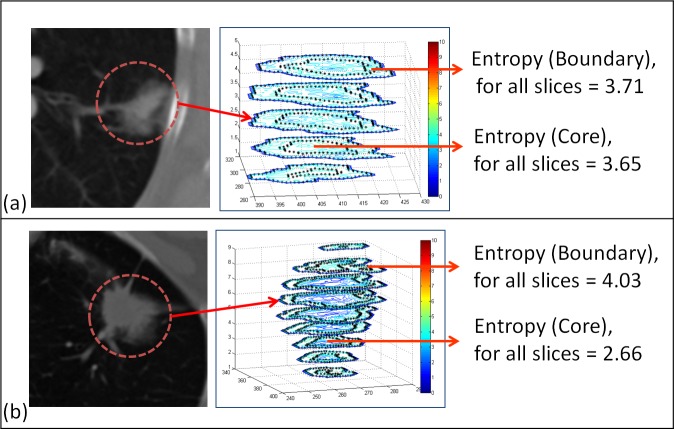
Entropy ratio was developed to quantify intensity variations across the tumor. While some tumors present with consistent mean entropy across the core and the boundary (a), others have a distinct difference in the values (b).

Prior to entropy filtering, each CT slice containing tumor was converted to its normalized grayscale equivalent. Binning Hounsfield units into 256 discrete intensity bins allowed the method to normalize tumor intensity ranges and to emphasize the differences in intensity gradient over small local pixel intensity variations. For each pixel within the tumor, an entropy coefficient was computed in its 7-by-7 pixel surrounding neighborhood. The mean of the coefficients was computed for pixels in the boundary and core regions as ensemble scores to represent them (Fig. 2). Entropy ratio feature was computed by dividing entropy score of the boundary region by the entropy score of the core region in order to characterize the contrast between them.

**Image feature reproducibility analysis.** Since feature stability is an important trait for a biomarker, we tested the developed metrics against typical patient variations using the Reference Image Database to Evaluate Therapy Response (RIDER) dataset. The RIDER project was a National Cancer Center (NCI) sponsored project to create consensus across institutions and help in harmonization of the quantitative features. The dataset was downloaded from the NBIA National Biomedical Imaging Archive [18]. Unenhanced thoracic CT images for 32 patients in the test-retest dataset (baseline and follow-up) were acquired within 15 minutes of each other, using identical CT scanner and imaging protocol[18]. GE Medical Systems Lightspeed 16 or VCT scanner with 16/ 64 detectors in 28/4 patients were used, respectively. The dataset was matched with equal number of early and late stage tumors with equal mixture of men and women in the study.

Tumors were delineated by a semi-automated segmentation tool with boundary markings finalized by a radiologist. Convexity and entropy ratio features were extracted independently from baseline and follow-up scans of the RIDER dataset and consistency of the assigned score was examined. For each proposed feature, concordance correlation coefficient (CCC) was computed to quantify reproducibility between two scans performed on each patient. The concordance correlation coefficient measures deviation from the 45 degree line which has been shown to be superior to the Pearson correlation coefficient for comparing repeated experiments[19]. We then computed the Dynamic Range (DR) for each feature, which is defined as the average difference between measurements to the observed biological (inter-patient) range in the data set. The method has been first proposed and used to find informative feature set [20].

### Statistical analyses

The imaging feature data, demographic information, and vital status data were merged into a single file for subsequent statistical analyses using Stata/MP 12.1 (StataCorp LP, College Station, TX). Student’s t-test and ANOVA were used to test for differences in imaging features by the demographic features and imaging parameters. A correlation matrix was used to assess dependency between the imaging features. Survival analyses were performed using Cox proportional hazard regression and Kaplan-Meier survival curves; statistical significance was computed using the log-rank test. The imaging features were dichotomized into two groups using the median score value.

## Results

### Demographics and imaging parameters by imaging biomarkers

Table 1 captures the variability of key clinical and imaging parameters for Cohort 1. This variability is representative of clinical applications that rely on patient imaging captured during the course of clinical care rather than for research purposes.

**Table 1:** Distribution of study population demographics and imaging parameters by imaging biomarkers in Cohort 1.

			Imaging biomarkers					
			Entropy ratio		Tumor volume		Convexity	
Characteristic ^1^	No.	(%)	Mean	(SD)	Mean	(SD)	Mean	(SD)
**Overall**	61	(100.0)	1.41	(0.26)	7884.6	(11205.9)	0.87	(0.07)
**Demographics**								
***Age at diagnosis***								
< 65	20	(32.8)	1.42	(0.05)	11436.4	(3651.2)	0.87	(0.02)
≥ 65	41	(67.2)	1.40	(0.04)	6152.0	(1129.7)	0.86	(0.01)
P-value			0.834		0.084		0.829	
***Gender***								
Female	30	(49.2)	1.35	(0.04)	6467.9	(1462.0)	0.87	(0.1)
Male	31	(50.8)	1.46	(0.05)	9255.6	(2444.3)	0.87	(0.1)
P-value			0.102		0.336		0.995	
***Stage***								
Stage I	25	(41.0)	1.36	(0.27)	5875.2	(10830.4)	0.87	(0.07)
Stage II	19	(31.2)	1.44	(0.24)	9791.5	(10927.1)	0.86	(0.09)
Stages III and IV	17	(27.8)	1.42	(0.25)	8708.2	(12217.9)	0.87	(0.07)
P-value			0.590		0.493		0.817	
**Imaging Parameters**								
***Voltage, KvP***								
120	57	(93.4)	1.42	(0.26)	8187.2	(11510.8)	0.87	(0.08)
130 or 140	4	(6.6)	1.22	(0.19)	3572.3	(3406.5)	0.85	(0.03)
P-value			0.124		0.431		0.761	
***Convolution kernel***								
B30F	8	(13.1)	1.47	(0.24)	3314.3	(4500.1)	0.845	(0.13)
B40f	19	(31.2)	1.31	(0.15)	11752.4	1(5712.3)	0.837	(0.07)
B41F	22	(36.1)	1.46	(0.25)	6487.3	(7959.1)	0.889	(0.05)
Other^2^	12	(19.7)	1.44	0.37)	7369.0	(10095.2)	0.884	(0.08)
P-value			0.222		0.269		0.101	
***Slice thickness, mm***								
<5.0	18	(29.5)	1.47	(0.26)	9836.8		0.844	(0.10)
≥ 5.0	43	(70.5)	1.38	(0.25)	7067.4	(11301.7)	0.875	(0.06)
P-value			0.181		0.383		0.142	
***Pixel resolution*^*3*^, *mm***								
< 0.6926	20	(32.8)	1.44	(0.25)	8013.6	(10733.4)	0.875	(0.06)
≥ 0.6926 to < 0.7785	20	(32.8)	1.36	(0.21)	10414.8	(14356.6)	0.878	(0.09)
≥ 0.7785	21	(34.4)	1.49	(0.29)	5352.1	(7636.5)	0.845	(0.09)
P-value			0.529		0.357		0.290	

^1^ 96.7% (No. = 59) of this study population were ever smokers and 96.7% (No. = 59) were White race

^2^ Other includes B30s, B41s, B70s, CHST, FC01, FC13, LUNG, and STANDARD

^3^ Distribution based on the tertile values

There were no statistically significant differences in the imaging biomarkers by age, gender, stage, or by the imaging parameters including KVP, convolution kernel, slice thickness, or pixel resolution (Table 1). We however observed significant differences for both imaging features by stage (Convexity: p = 0.017; Entropy Ratio: p = 0.002) and for entropy ratio feature, by convolution kernel (p = 0.026) in Cohort 2 (Table A in S1 File).

### Convexity and Entropy Ratio features reproducibility confirmed by Test-Retest analyses

Concordance correlation coefficients (CCC) and dynamic range (DR), averaged over the volume of the tumor, are summarized in Table B in S1 File. CCCs and DR for the features extracted from a single center slice of the tumor, matched by a radiologist between test and retest scans, are summarized in Table C in S1 File. Convexity feature showed high concordance in the test-retest experiment (>0.88) in both single slice and volumetric evaluations. Entropy ratio had a low CCC when averaged over the volume of the tumor. While entropy of the core and boundary regions had high CCC (>0.81), the concordance metric penalized their ratio as entropy ratio scores showed unproportional variation to the 45° line. The third column of Table B in S1 File shows the statistics of the absolute percent difference between test and retest feature values and it should be noted that the mean difference for entropy ratio feature is approximately 1.69%. Both convexity and entropy ratio showed high level of concordance (>0.99) and dynamic range (>0.96) in single slice evaluation (Table C in S1 File).

The repeatability of the features confirms their ability to consistently score tumor characteristics with respect to variability in the repeated experiments (S4 Fig.). The tumors in the RIDER set are a diverse population of mixed stages and histology. The Bland-Altman plot for the computed features (S5 Fig.) shows close bound (95% confidence) for the individual cases; larger test-retest variations can be attributed to the above discussed challenges.

### Overall Survival of Convexity Feature

A lower convexity score was reflective of a more irregular shape and expected worse survival. Higher convexity scores describe convex shapes with fewer irregularities along the boundary. Fig. 1B demonstrates tumor shape morphology ordered according to the computed convexity score. Fig. 1C shows that high (> median) and low convexity separated patients with good and poor overall survival time (p = 0.008). Convexity values for the Cohort 1 ranged from 0.57 to 0.97, with the median value = 0.89.

### Overall Survival of Entropy Ratio Feature

**Fig 3:**
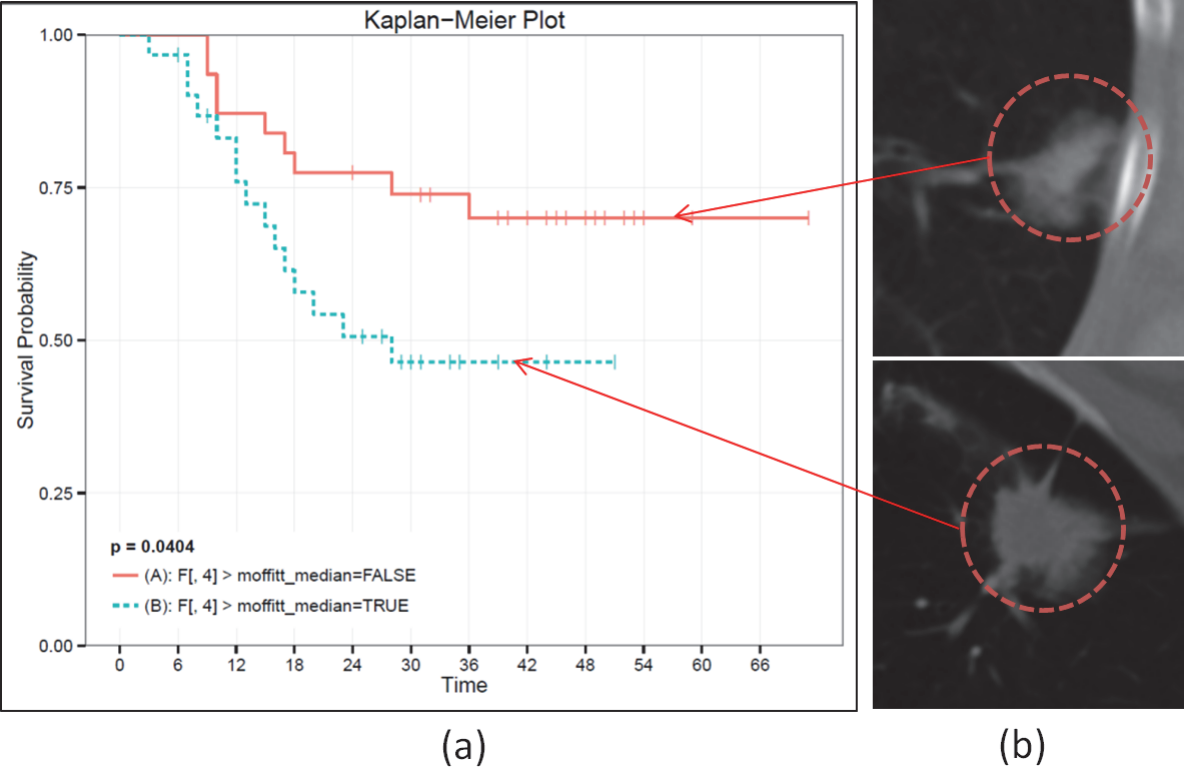
Entropy ratio between the core and border regions of the tumor is predictive of patient survival. The tumors in the two prognostic groups (a) did not appear significantly different in the CT scans (b).

Median score were used to discriminate between low and high entropy groups. Entropy measures for the entire segmented region were not statistically significant (p = 0.28) with respect to overall survival. Evaluating entropy within the boundary region only was also not significant (p = 0.96). Furthermore, it appeared that high entropy values in the border region skewed the performance of the feature when calculated across the entire tumor region. While restricting the calculation of entropy to the core region was not statistically significant (p = 0.059), tumors with high (>median) core entropy tended to associate with worse overall survival. Entropy of the core may capture important intratumor characteristics such as necrosis and heterogeneity. While entropies of core and boundary regions were not statistically significant independently, their ratio was associated with overall survival (p = 0.04, Fig. 3A). Tumors for which the entropy was consistent throughout the core and boundary regions (i.e. ratio<1.41; S6 Fig.) were associated with better survival outcome and tumors presenting with larger (>1.41) ratios (S6 Fig.) were correlated with worse overall survival.

Representative CT slices of the tumors from the two prognostic groups, as defined by larger than median entropy ratio of the core and boundary regions are shown in Fig. 3B. When visually inspected by resident radiologists, tumors from these two prognostic groups were not described as being prognostically different. In fact, the tumor at the top in Fig. 3B might be considered to have worse prognosis due to its attachment to the pleural wall[21], which is a known negative prognostic indicator.

### Cox Proportional Hazards Models

When the imaging biomarkers were analyzed independently in separate univariate models for overall survival (Table 2) in Cohort 1, we noted statistically significant associations with tumor volume (HR = 2.59; 95% CI 1.06–6.29) and convexity (HR = 0.34; 95% CI 0.14–0.82), and a borderline significant association with entropy ratio (HR = 2.19; 95% CI 0.94–5.08). Age, gender, and stage were significantly associated with survival in the univariable models. When all three imaging biomarkers were included in a stepwise forward selection model (see methods), tumor volume dropped out while convexity (HR = 0.32; 95% CI 0.13–0.78) and entropy ratio (HR = 2.33; 95% CI 1.00–5.45) remained. Convexity and entropy ratio remained statistically significant in the final multivariable model adjusting for age, gender, and stage. The final model was replicated in Cohort 2. Although the hazard ratios for entropy and convexity were not statistically significant, the point estimates trended in the same direction.

**Table 2:** Cox Proportional Hazards Models for Overall Survival.

	Cohort 1 (N = 62)						Cohort 2 (N = 47)	
Covariates^1^	Unadjusted HR (95% CI)^2^	P-value	Multivariable HR (95% CI)^3^	P-value	Multivariable HR (95% CI)^4^	P-value	Multivariable HR (95% CI)^4^	P-value
**Entropy ratio**	2.19 (0.94–5.08)	0.07	**2.33 (1.00–5.45)**	**0.05**	**2.36 (1.00–5.58)**	**0.05**	1.24 (0.53–2.87)	0.62
**Tumor volume**	**2.59 (1.06–6.29)**	**0.04**	—	—	—	—	—	—
**Convexity**	**0.34 (0.14–0.82)**	**0.02**	**0.32 (0.13–0.78)**	**0.01**	**0.31 (0.12–0.78)**	**0.01**	0.82 (0.33–2.03)	0.67
**Age**	1.22 (0.50–2.96)	0.67	—	—	1.11 (0.44–2.83)	0.82	1.54 (0.60–3.97)	0.36
**Gender**	1.25 (0.55–2.85)	0.60	—	—	1.76 (0.73–4.23)	0.21	1.49 (0.65–3.46)	0.35
**Stage**	1.52 (0.92–2.53)	0.11	—	—	1.45 (0.84–2.49)	0.18	**2.73 (1.36–5.48)**	**< 0.01**

Abbreviations: Hazard Ratio, HR; Confidence Intervals, CI.

Statistically significant hazard ratios (p < 0.05) are shown in bold.

^1^ The imaging features are dichotomized at their respective median values and age is dichotomized at 65 years

^2^ Each imaging biomarker is analyzed independently in separate univariate models. The unadjusted HRs represent the main effects of each covariate.

^3^ Based on forward selection, only two imaging biomarkers are included in the model but excluded age, gender, and stage.

^4^ Only two imaging biomarkers are included in the model in addition to age, gender, and stage

### Imaging features s performance in an independent cohort

**Fig 4:**
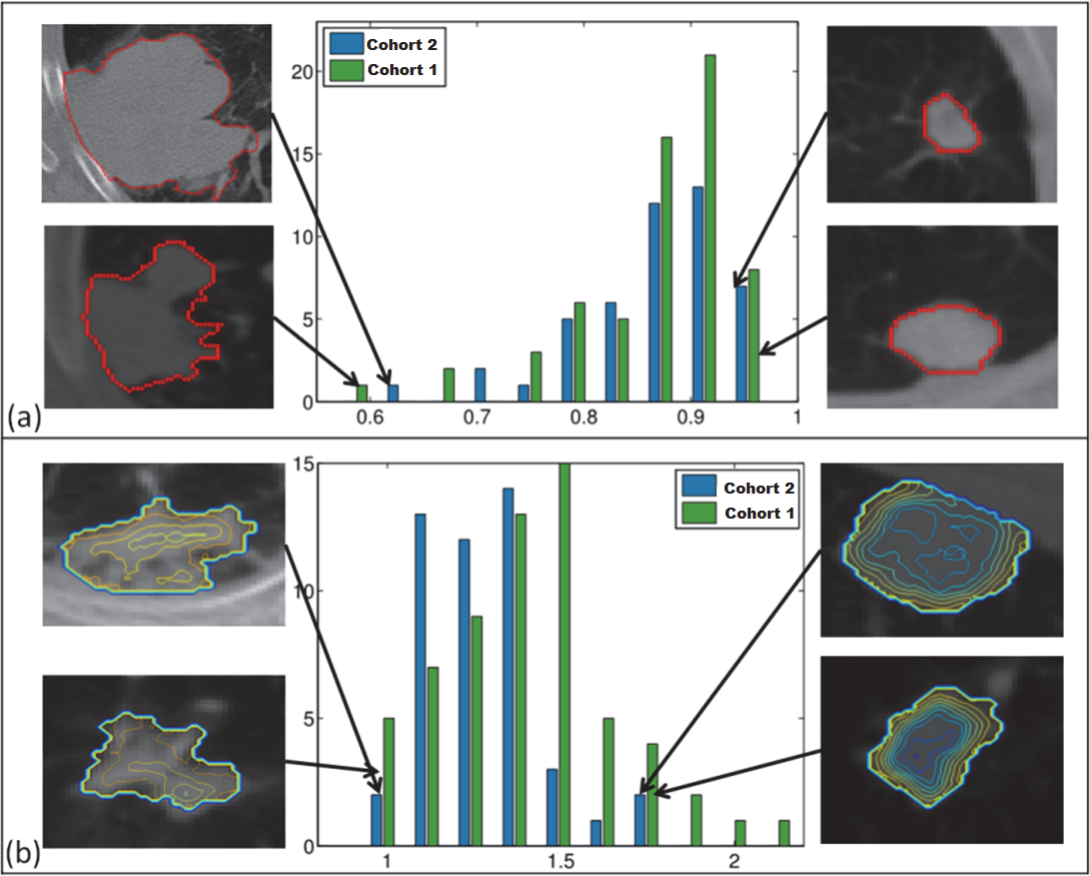
Histogram of the two imaging features across cohorts. Convexity (a) shows similar range across cohorts (training-green, test-blue) However, training cohort is enriched with round tumors. The range of values for entropy ratio feature (b) is larger in training cohort. Both convexity (a) and entropy ratio (b) consistently capture targeted tumor characteristics in both cohorts.

The distributions of the computed feature scores were compared between two independent cohorts: Cohort 1 and Cohort 2 (Fig. 4). The convexity descriptor had a similar range in both cohorts (Fig. 4A); however, Cohort 1 (green) distribution was skewed towards rounder and more convex tumor shapes. In either of the cohorts, low convexity corresponded to more irregular tumor shapes, while high convexity scores were indicative of rounder shapes with fewer concavities and irregularities along the tumor perimeter.

Histograms of entropy ratio scores were different between the cohorts (Fig. 4B). Contour plots of entropy coefficients in the tumor regions were generated to visualize intratumor entropy differences in extreme imaging phenotypes. The phenotypes described by the low value entropy ratio (Fig. 4B, left) were similar across both cohorts. Contour plot profile corresponding to high entropy ratio (Fig. 4B, right) was consistent for both cohorts, as well.

Survival analyses were carried out on Cohort 2 strictly in accordance with the previously established procedure for Cohort 1. This is a very stringent test of reproducibility, as the populations and acquisition conditions were different between the cohorts. Based on the median splits derived from Cohort 1 (convexity = 0.89, and entropy ratio = 1.4), in Cohort 2 neither convexity (p = 0.7) nor entropy ratio (p = 0.8) were associated with overall survival. In Cohort 2, convexity feature was statistically significant with respect to survival (p = 0.008) in univariate Cox proportional hazards regression analysis.

The developed tumor descriptors consistently and objectively scored and ordered tumors according to their shape (S7 Fig.) and intratumor intensity variation (S8 Fig.) in both datasets.

## Discussion

The aim of this study was to develop objective and robust quantitative imaging descriptors that were associated with patient survival. The developed CT features quantified lung adenocarcinomas based on their shape and intratumor intensity variations. The features systematically scored tumors and identified imaging phenotypes which exhibited survival differences in two independent cohorts. The features were extracted from routinely obtained CT images and were reproducible and stable despite the inherent clinical image acquisition variability.

Outcomes after resection in early-stage NSCLC are poor, with 30–50% of the deaths due to distant recurrence[22]. Current decision support for the use of adjuvant chemotherapy following surgery is ill-informed. Gene signatures [2] have been developed to improve risk stratification of early stage NSCLC patients and while an improvement, the approach suffers from sampling bias, as to all biopsy-dependent approaches. Incorporating additional knowledge from individual patients’ scans into the post-operative decision making can potentially identify distinct phenotypes of the disease and serve as a noninvasive approach to identify high-risk patients who may benefit from additional post-surgical treatment such as adjuvant chemotherapy. In addition, imaging features can be used as surrogates for tumor specific factors when limited pathological specimens are obtained from diagnostic biopsies [21]. Furthermore, we believe that inferring tumor biological activity and aggressiveness from minimally-invasive imaging techniques can inform precision medicine strategies longitudinally where serial biopsies may be too difficult to achieve.

In order to avoid data sparsity, we recognized the importance of keeping the number of imaging features reasonably low and reflective of the cohort. It has been recommended to keep a rather stringent ratio between the number of patients in each prognostic class and the features above 10[23]. In order to adequately relay tumor characteristics while coping with dimensionality restrictions, preference should be given to hypothesis-driven features predicated on available biomarkers and tumor biology associations[24].

Owning to the absence of a validation cohort with matching clinical and imaging parameters, we could not adequately explore the prognostic behavior of the developed features. Validation of imaging features derived from retrospectively assembled cohorts presents an interesting challenge as the field continues to mature. It is essential to refute spurious findings by validating imaging biomarkers in independent, previously unseen cohorts. However, the lack of ‘golden standard’ for testing derived features is often coupled with a number of confounding factors stemming from imaging variability and heterogeneity within patient cohort. It is therefore important to recognize that differences in patient population, quality and manner of data acquisition and subsequent processing can result in validation cohorts that are not adequate for fair assessment. This also highlights the significance and merits of hypothesis-driven imaging feature design which can serve as additional validation criteria in an independent cohort. In our study we have taken the effort to develop image based metrics to quantify observable tumor characteristics.

Additional exploration of the prognostic utility was carried out in an independent cohort (Cohort 2) from a partnering institution located on a different continent, with inherent differences in patient population. In both cohorts, extreme tumor phenotypes were identified based on the distribution of the computed feature scores (Table D in S1 File). Patient subpopulations were chosen using the opposite extreme quartiles of the convexity score distribution (<0.8 and >0.9) from Cohort 1. Survival difference between the two phenotypes was statistically significant for Cohort 2 (p = 0.02; Cohort 1: p = 0.06). Similarly, two subpopulations were identified using low (<1.23) and high (>1.5) entropy ratio scores from Cohort 1. The difference between the subpopulations was statistically significant for Cohort 1 (15 patients vs. 19 patients, p = 0.04). However, due to the differences in entropy score distributions (Fig. 4B), there were not enough patients (n = 2) in Cohort 2 to represent the phenotype with high (>1.5) entropy ratio scores. The survival significance found in the entire Cohort 1 and in Cohort 2, using quartile split, is promising.

Our study had several limitations. The cohort sample sizes were small which limited our ability to perform extensive stratified analyses. Additionally, the two cohorts are likely not comparable since we have observed significant differences for the imaging features by stage and convolution kernel in Cohort 2 but no difference by stage (or any other characteristic) in Cohort 1. While patients in Cohort 1 were surgical candidates, predominantly stages I and II, Cohort 2 consisted of radiotherapy planning patients with more advanced stages of the disease. Furthermore, the overall survival trend for the cohorts differed (S9 Fig.). The differences between the two cohorts may explain why our multivariate model did not replicate in Cohort 2 (Table 2). Standardization of image protocols remains a challenge in image biomarker development[25]. Variability in image acquisition and reconstruction parameters is inherent to retrospective imaging studies. Although this variability can be a significant limitation in studies, ultimately for the computed tomographic biomarkers to be adopted into clinical practice and utilized across multiple imaging centers, these biomarkers must be stable in the presence of image acquisition variability[26]. The size of our cohorts and imaging parameter variability are equivalent to other recently published studies [7,13,27,28]. The strength of our study was the development of imaging features that were descriptive and reproducible using retrospectively acquired clinical scans.

Our results suggest that quantitative imaging biomarkers can be used as an additional diagnostic tool in management of lung adenocarcinomas. Although more work is needed to determine clinical utility, it is clear that these descriptors are capable of quantifying and consistently ranking key tumor characteristics. Imaging biomarkers, combined with RECIST measurements and laboratory test results, will in the future be the *de facto* standard of a decision support pipeline employed to personalize and optimize treatment protocols.

## Supporting Information

S1 Fig Flowcharts were used to visualize the algorithms for the calculated features. Convexity (a) and entropy ratio (b) algorithms are described.
(TIF)

S2 Fig Automatic detection of tumor attachment to pleural wall was carried out by comparing the perimeter of the lung with tumor perimeter. The lung perimeter is outlined with green unfilled circles and tumor perimeter—with red filled circles.
(TIF)

S3 Fig Segmented tumor region is subdivided in order to quantify intratumor variation of entropy. Original tumor segmented ROI (a) is subdivided into core and boundary sub-regions (c). Tumor ROI (b, red) is dilated (b, pink) and eroded (b, green). Subtracting the core mask (b, bottom, green contour) from the dilated region forms the boundary mask (b, bottom, pink contour).
(TIF)

S4 Fig Tumor perimeters from RIDER Lung test-retest dataset were segmented using Ensemble algorithm. Representative slices from tumors with high (a,b) and low (c,d) convexity scores are displayed. Tumor scores were computed on baseline scans: 0.54(a) and 0.92(c) and follow-up scans: 0.6 (c) and 0.91 (d).
(TIF)

S5 Fig Bland-Altman plot for the CT features (convexity, entropy of the tumor core, entropy ratio and entropy of the tumor boundary) demonstrates individual variability in the test-retest experiment against the average measurement. The dotted lines show 95% confidence limit for the features in the sample set.
(TIF)

S6 Fig Contour plots of entropy coefficients were used to visualize their intratumor variation. Two classes of tumors were identified: tumors with low entropy ratio between core and boundary regions (a) and tumors with high entropy ratio between core and boundary regions (b).
(TIF)

S7 Fig Tumors were ordered based on their convexity score and show a progression from irregular to convex shape with the increasing score. Convexity feature consistently scored tumor shape in both cohorts (Cohort 1: a; Cohort 2: b).
(TIF)

S8 Fig Color graphs were used to visualize entropy coefficients: their range was divided into four sub-ranges and assigned a unique color. Tumors were ordered based on their entropy ratio score and showed similar patterns of entropy coefficient distribution in both cohorts. Entropy ratio feature consistently scored intratumor density variation in both cohorts (Cohort 1: a; Cohort 2: b).
(TIF)

S9 Fig Comparing survival trends between two cohorts. Overall survival trends for Cohort1 (a) and Cohort 2 (b) are different.
(TIF)

S1 File Table A, Distribution of study population demographics and imaging parameters by imaging biomarkers in Cohort 2. Table B, Concordance correlation coefficient (CCC) and dynamic range for CT features in the test-retest experiment, averages over tumor volume. Table C, Feature Reproducibility in test-retest analyses using matching center slice on the CT image. Table D, Clinical parameters and feature scores for Cohort 1 patients.
(DOCX)
