# Correction: Quantitative Computed Tomographic Descriptors Associate Tumor Shape Complexity and Intratumor Heterogeneity with Prognosis in Lung Adenocarcinoma

**DOI:** 10.1371/journal.pone.0248541

**Published:** 2021-03-08

**Authors:** Olya Grove, Anders E. Berglund, Matthew B. Schabath, Hugo J. W. L. Aerts, Andre Dekker, Hua Wang, Emmanuel Rios Velazquez, Philippe Lambin, Yuhua Gu, Yoganand Balagurunathan, Edward Eikman, Robert A. Gatenby, Steven Eschrich, Robert J. Gillies

The Data Availability statement is being updated with a new data location. The updated and correct statement is: Quantitative features data for Cohort 1 has been added to Suppl. materials. Cohort 1 deidentified image data will be available via TCIA, upon publication. The dataset can be found at: https://public.cancerimagingarchive.net/ncia/login.jsf Using Search, under Collection(s), the dataset is available as LungCT-Diagnosis, and the DOI is https://doi.org/10.7937/K9/TCIA.2015.A6V7JIWX. Patient scan and clinical data for Cohort 2 is de-identified and can be made available upon request. Please contact Olya.Grove@moffitt.org, to establish a data transfer protocol, for images.
