# Supplementary figures and images for: Quantitative Computed Tomographic Descriptors Associate Tumor Shape Complexity and Intratumor Heterogeneity with Prognosis in Lung Adenocarcinoma
Source: PLoS One. 2015 Mar 4;10(3):e0118261. doi: 10.1371/journal.pone.0118261 (PMC4349806; doi:10.1371/journal.pone.0118261)

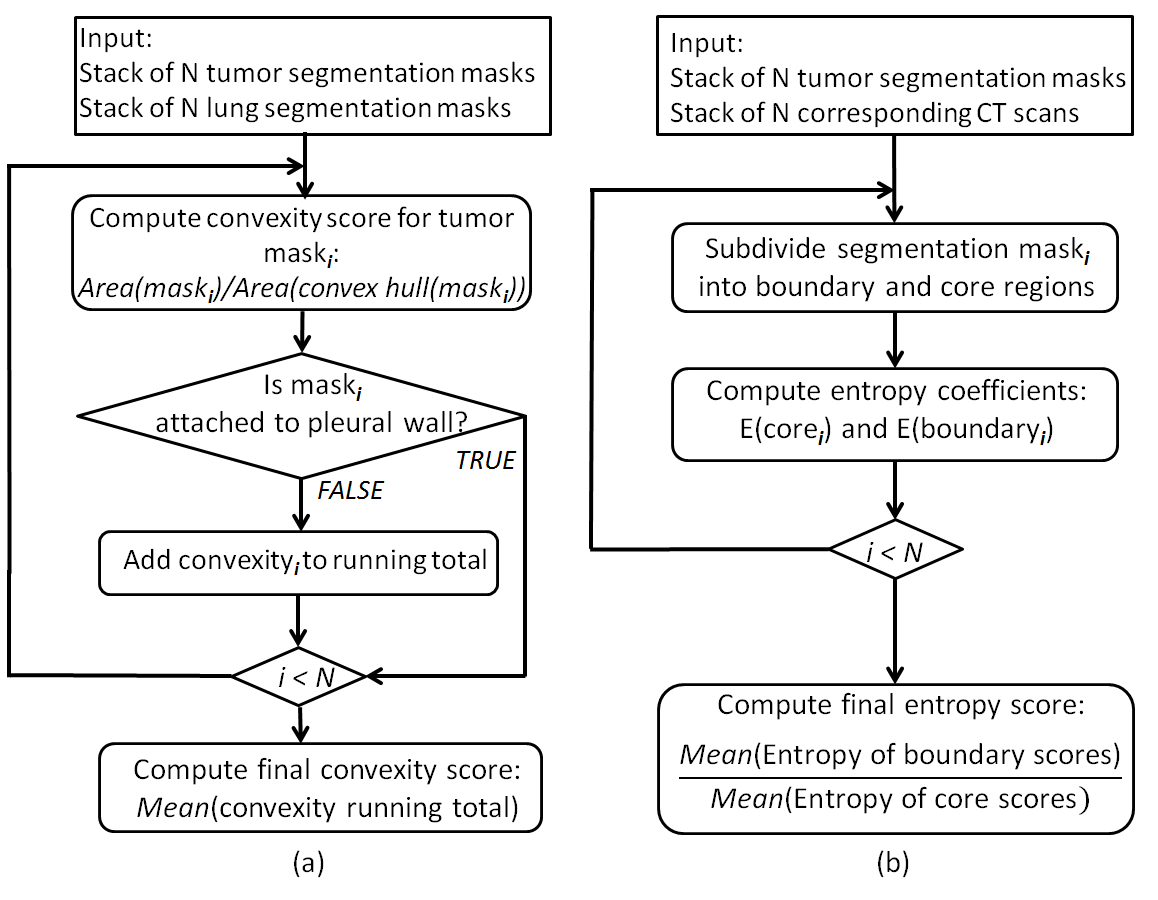

Supplement: S1 Fig — Convexity (a) and entropy ratio (b) algorithms are described. (TIF) [file pone.0118261.s001.tif]

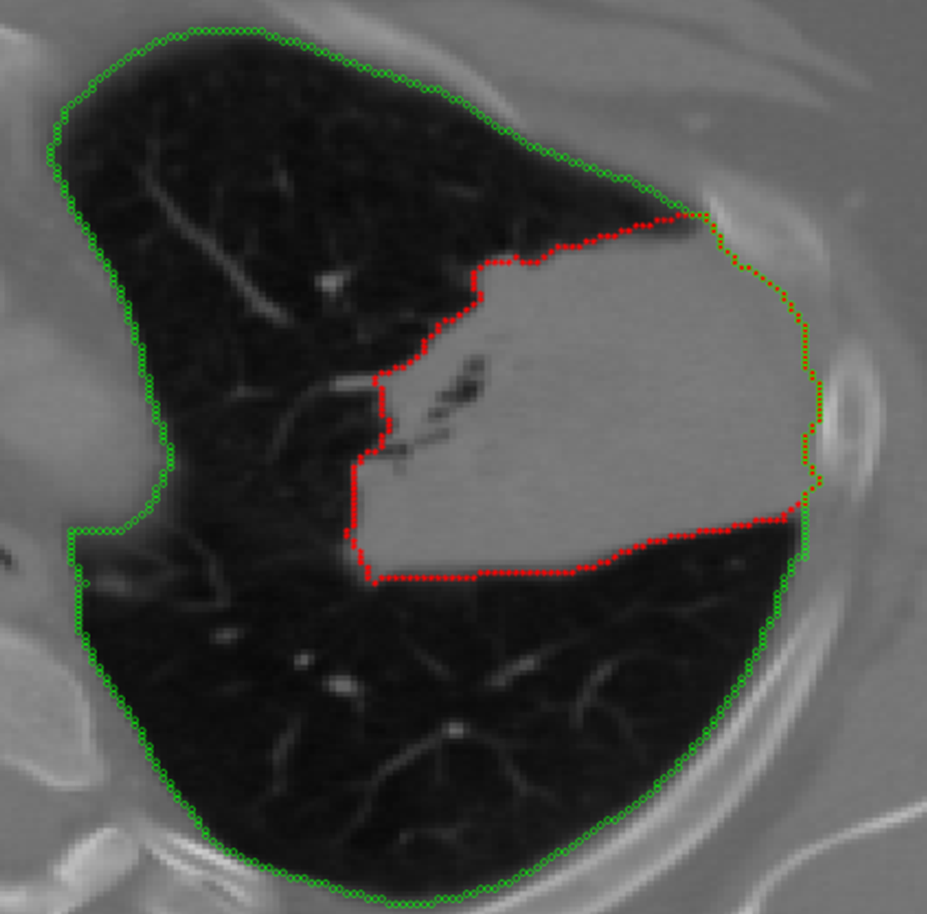

Supplement: S2 Fig — The lung perimeter is outlined with green unfilled circles and tumor perimeter—with red filled circles. (TIF) [file pone.0118261.s002.tif]

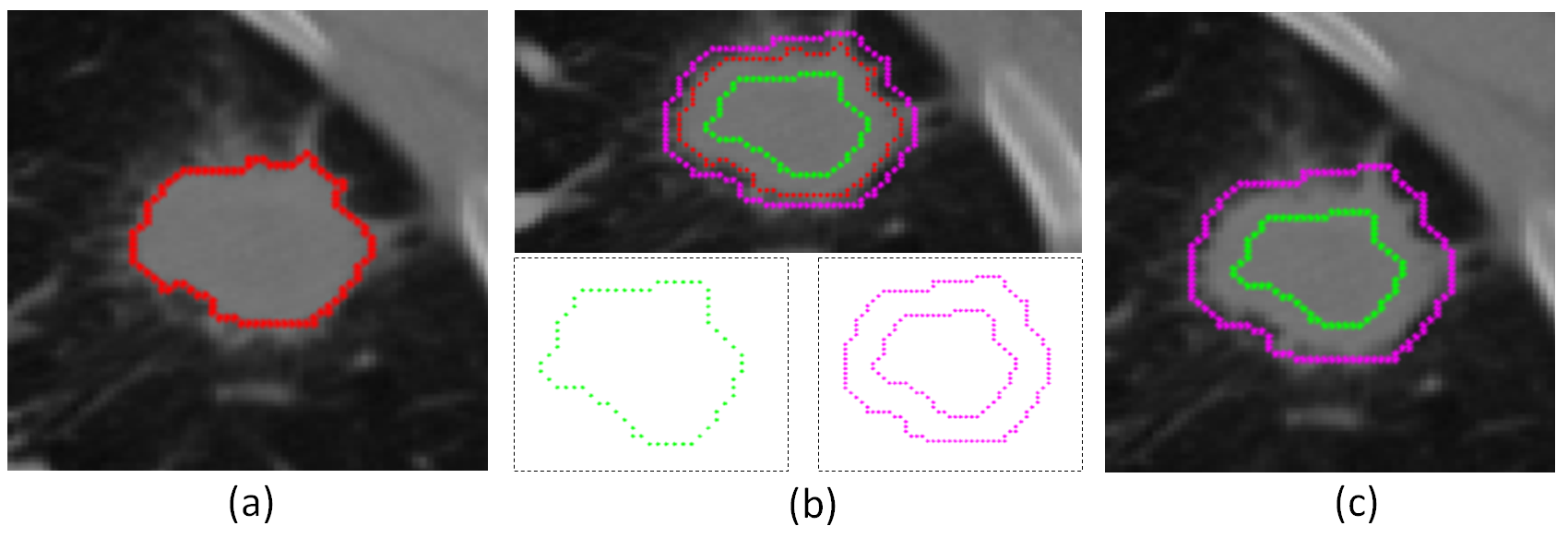

Supplement: S3 Fig — Original tumor segmented ROI (a) is subdivided into core and boundary sub-regions (c). Tumor ROI (b, red) is dilated (b, pink) and eroded (b, green). Subtracting the core mask (b, bottom, green contour) from the dilated region forms the boundary mask (b, bottom, pink contour). (TIF) [file pone.0118261.s003.tif]

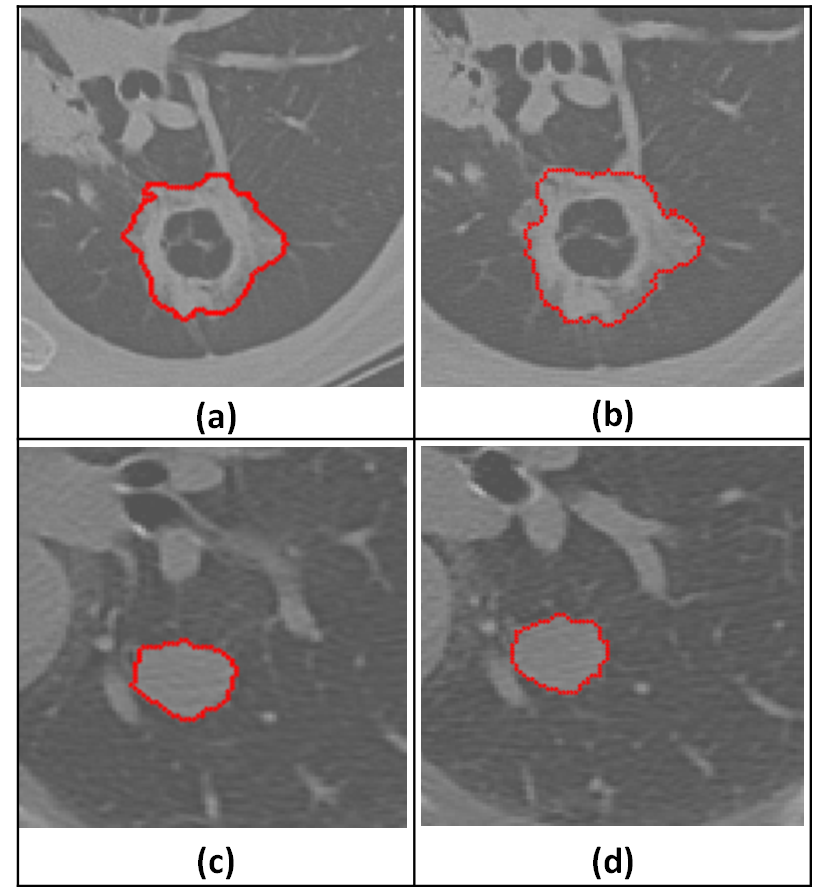

Supplement: S4 Fig — Representative slices from tumors with high (a,b) and low (c,d) convexity scores are displayed. Tumor scores were computed on baseline scans: 0.54(a) and 0.92(c) and follow-up scans: 0.6 (c) and 0.91 (d). (TIF) [file pone.0118261.s004.tif]

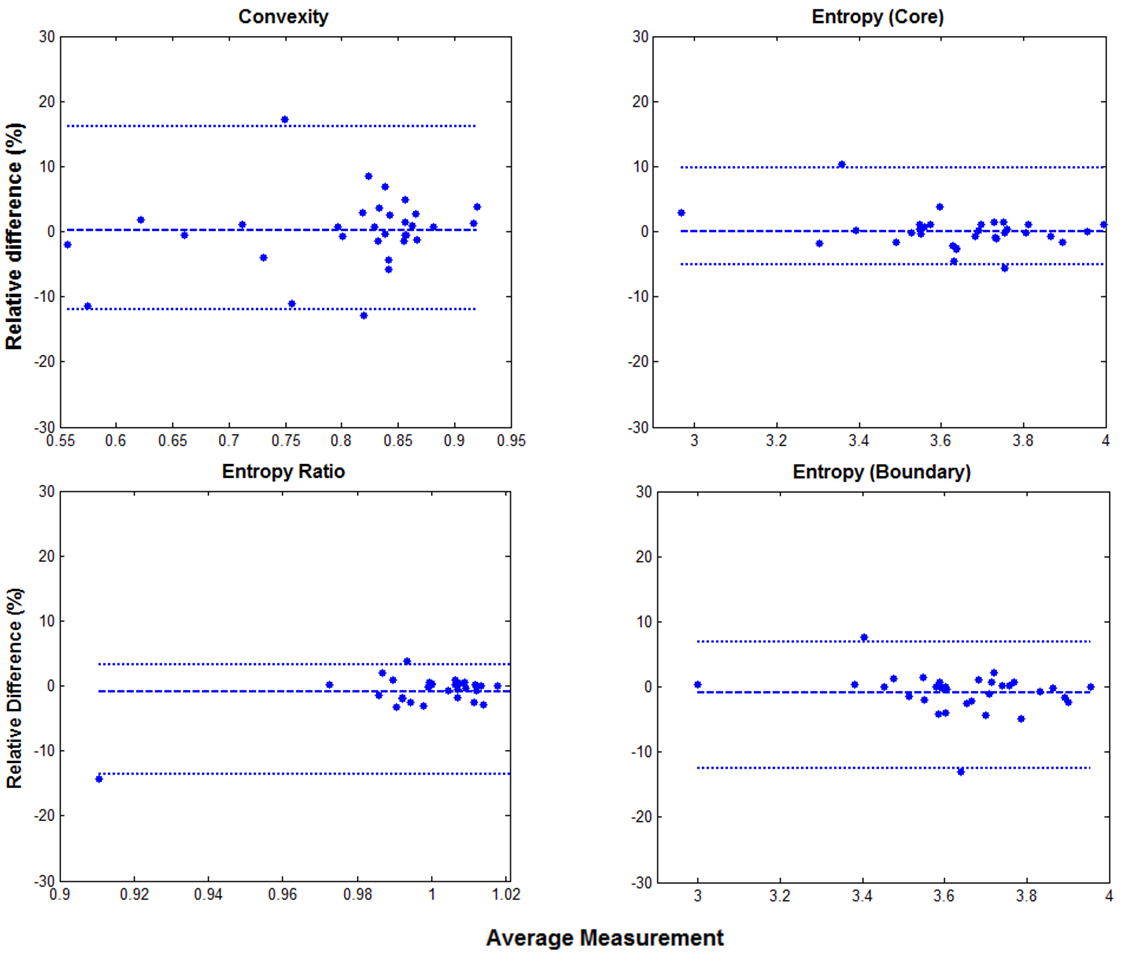

Supplement: S5 Fig — The dotted lines show 95% confidence limit for the features in the sample set. (TIF) [file pone.0118261.s005.tif]

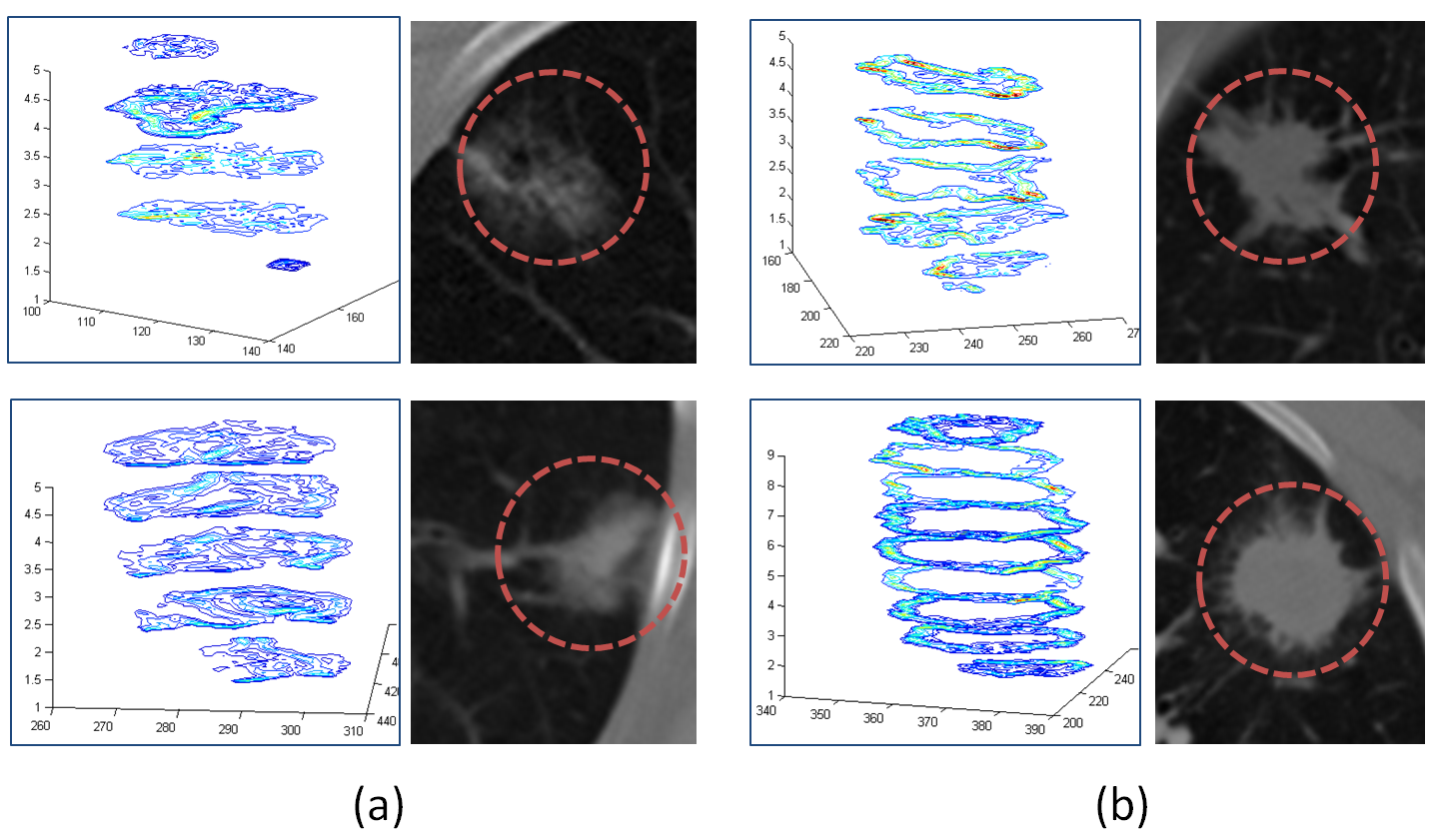

Supplement: S6 Fig — Two classes of tumors were identified: tumors with low entropy ratio between core and boundary regions (a) and tumors with high entropy ratio between core and boundary regions (b). (TIF) [file pone.0118261.s006.tif]

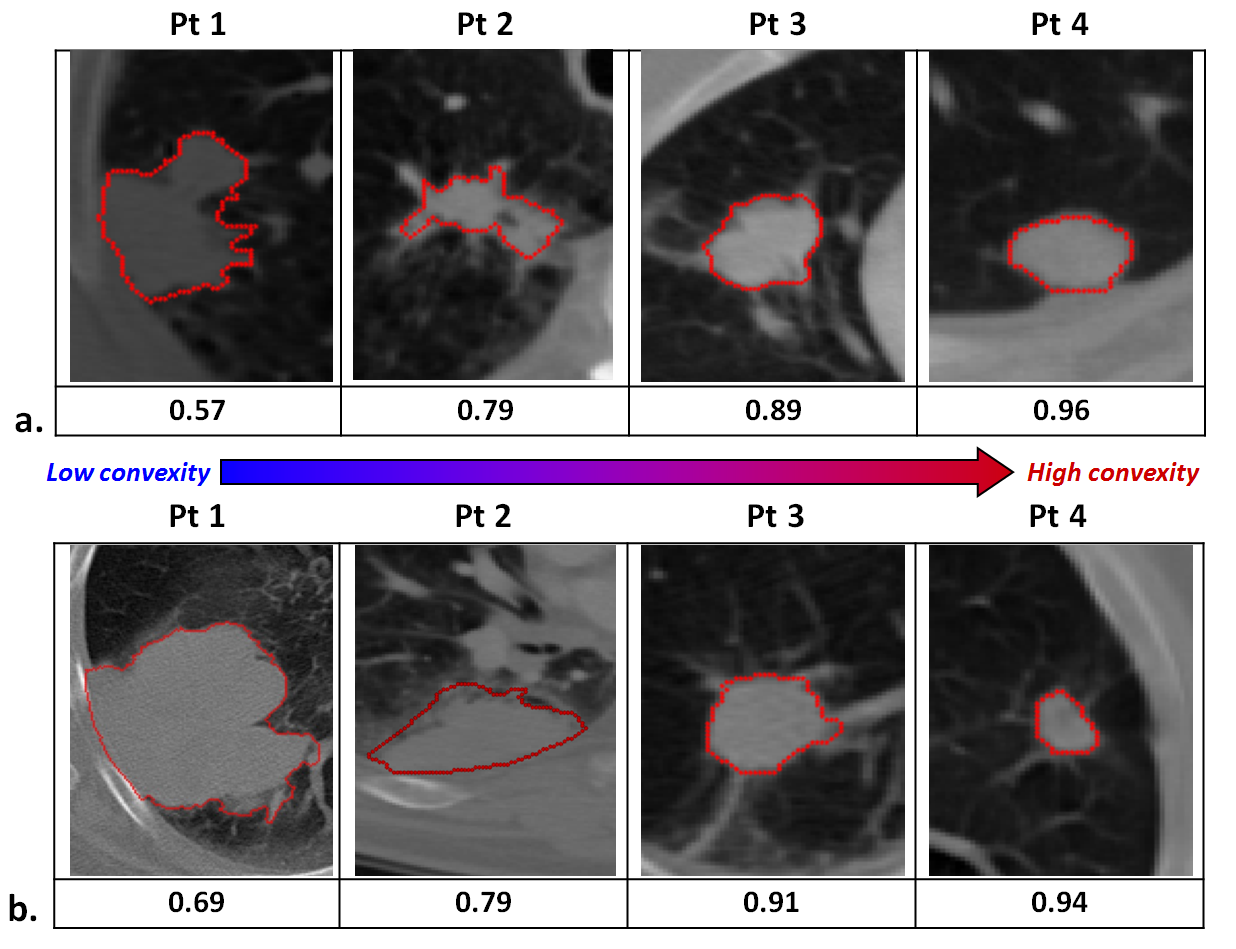

Supplement: S7 Fig — Convexity feature consistently scored tumor shape in both cohorts (Cohort 1: a; Cohort 2: b). (TIF) [file pone.0118261.s007.tif]

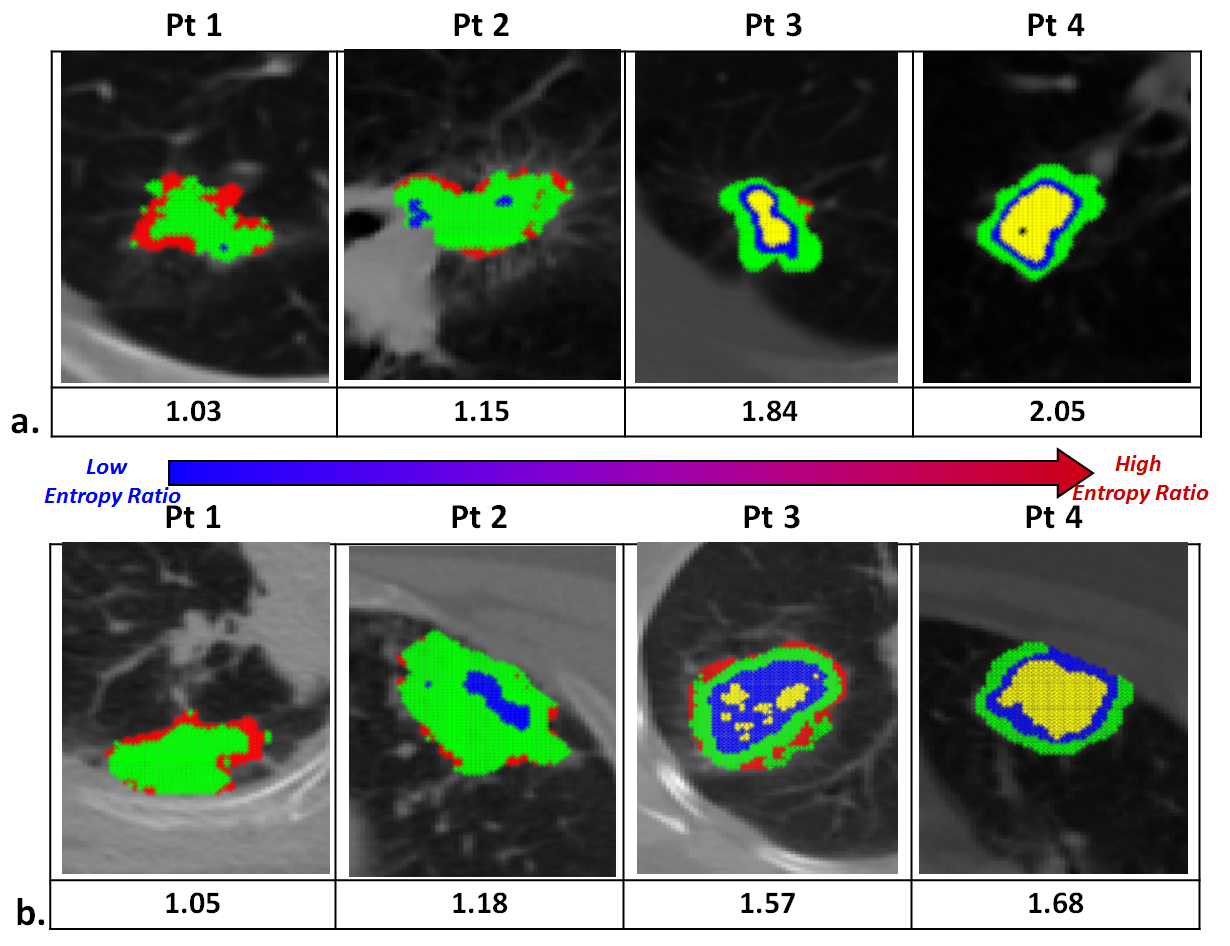

Supplement: S8 Fig — Tumors were ordered based on their entropy ratio score and showed similar patterns of entropy coefficient distribution in both cohorts. Entropy ratio feature consistently scored intratumor density variation in both cohorts (Cohort 1: a; Cohort 2: b). (TIF) [file pone.0118261.s008.tif]

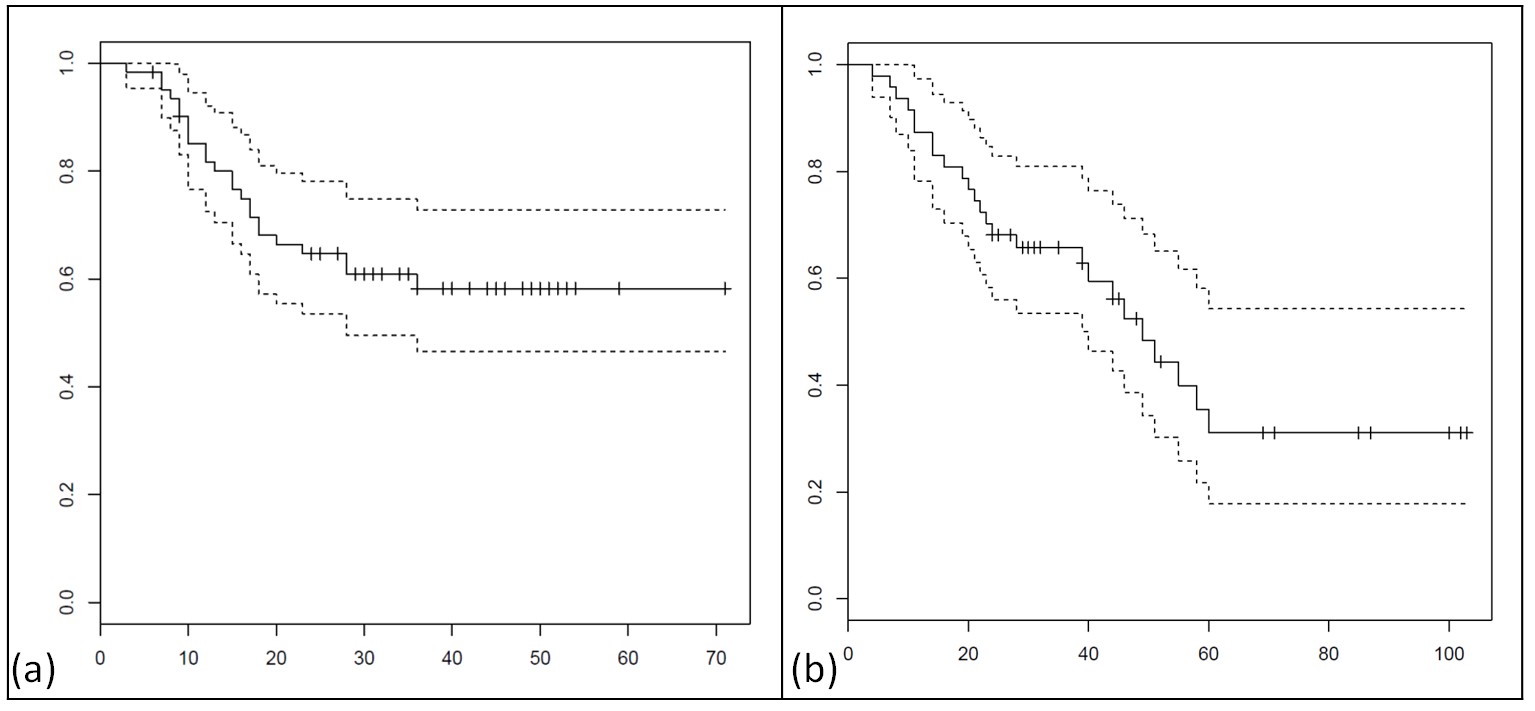

Supplement: S9 Fig — Overall survival trends for Cohort1 (a) and Cohort 2 (b) are different. (TIF) [file pone.0118261.s009.tif]
