# Supplementary material for: Quantitative Computed Tomographic Descriptors Associate Tumor Shape Complexity and Intratumor Heterogeneity with Prognosis in Lung Adenocarcinoma
Source: PLoS One. 2015 Mar 4;10(3):e0118261. doi: 10.1371/journal.pone.0118261 (PMC4349806; doi:10.1371/journal.pone.0118261)
Supplement: S1 File — Table B, Concordance correlation coefficient (CCC) and dynamic range for CT features in the test-retest experiment, averages over tumor volume. Table C, Feature Reproducibility in test-retest analyses using matching center slice on the CT image. Table D, Clinical parameters and feature scores for Cohort 1 patients. (DOCX) [file pone.0118261.s010.docx]

| **Table A. Distribution of study population demographics and imaging parameters by imaging biomarkers in Cohort 2.** | | | | | | | | | | |
| --- | --- | --- | --- | --- | --- | --- | --- | --- | --- | --- |
|  | | |  | | **Imaging biomarkers** | | | | | |
| **Characteristic ^1^** | | |  | | **Entropy ratio** | | **Tumor volume** | | **Convexity** | |
|  |  |  | **No.** | **(%)** | **Mean** | **(SD)** | **Mean** | **(SD)** | **Mean** | **(SD)** |
|  | | |  |  |  |  |  |  |  |  |
| **Overall** | | | 47 | (100) | 1.27 | (0.16) | 1,576,573 | (5,538,671) | 0.86 | (0.07) |
|  | | |  |  |  |  |  |  |  |  |
| **Demographics** | | |  |  |  |  |  |  |  |  |
|  | ***Age at diagnosis*** | |  |  |  |  |  |  |  |  |
|  | | < 65 | 19 | (40.4) | 1.29 | (0.17) | 1,600,656 | (5,324,588) | 0.86 | (0.06) |
|  | | ≥ 65 | 28 | (59.6) | 1.27 | (0.16) | 1,560,231 | (5,776,054) | 0.87 | (0.08) |
| P-value | | |  |  | 0.697 | | 0.981 | | 0.725 | |
|  | | |  |  |  |  |  |  |  |  |
|  | ***Gender*** | |  |  |  |  |  |  |  |  |
|  | | Female | 22 | (46.8) | 1.27 | (0.17) | 1,673,242 | (6,449,505) | 0.87 | (0.07) |
|  | | Male | 25 | (53.2) | 1.28 | (0.16) | 1,491,504 | (4,731,241) | 0.87 | 0(.07) |
| P-value | | |  |  | 0.847 | | 0.912 | | 0.947 | |
|  | | |  |  |  |  |  |  |  |  |
|  | ***Stage^1^*** | |  |  |  |  |  |  |  |  |
|  | | Stage I | 10 | (21.7) | 1.21 | (0.13) | 87,739 | (150,848) | 0.89 | (0.04) |
|  | | Stage II | 21 | (54.7) | 1.35 | (0.18) | 385,901 | (675,387) | 0.89 | (0.05) |
|  | | Stages III and IV | 15 | (32.6) | 1.23 | (0.13) | 4,340,499 | (9,383,981) | 0.82 | (0.09) |
| P-value | | |  |  | **0.017** | | 0.067 | | **0.002** | |
|  | | |  |  |  |  |  |  |  |  |
| **Imaging Parameters** | | |  |  |  |  |  |  |  |  |
|  | ***Voltage, KvP*** | |  |  |  |  |  |  |  |  |
|  | | 120 | 40 | (85.1) | 1.27 | (0.16) | 1,046,870 | (3,786,242) | 0.87 | (0.07) |
|  | | 130 or 140 | 7 | (14.9) | 1.27 | (0.17) | 4,603,446 | (1.14e+07) | 0.86 | (0.09) |
| P-value | | |  |  | 0.954 | | 0.118 | | 0.761 | |
|  | | |  |  |  |  |  |  |  |  |
|  | ***Convolution kernel*** | |  |  |  |  |  |  |  |  |
|  | | A, B | 23 | (48.9) | 1.27 | (0.13) | 1,486,610 | (4,834,356) | 0.86 | (0.07) |
|  | | B30s ,B60f, B70s | 5 | (10.6) | 1.15 | (0.04) | 6,194,466 | (1,3532,86) | 0.85 | (0.10) |
|  | | B40f | 15 | (31.9) | 1.28 | (0.18) | 478,889 | (1,556,613) | 0.88 | (0.06) |
|  | | Other | 4 | (8.5) | 1.47 | (0.19) | 437,803 | (376,848) | 0.88 | (0.06) |
| P-value | | |  |  | **0.026** | | 0.240 | | 0.740 | |
|  | | |  |  |  |  |  |  |  |  |
|  | ***Slice thickness, mm*** | |  |  |  |  |  |  |  |  |
|  | | <5.0 | 18 | (38.3) | 1.25 | (0.17) | 3350,823 | (8,667,079) | 0.85 | (0.09) |
|  | | ≥ 5.0 | 29 | (61.7) | 1.29 | (0.16) | 475,314 | (1,228,994) | 0.88 | (0.06) |
| P-value | | |  |  | 0.345 | | 0.084 | | 0.161 | |
|  | | |  |  |  |  |  |  |  |  |
|  | ***Pixel resolution^4^, mm*** | |  |  |  |  |  |  |  |  |
|  | | < 0.9547 | 15 | (31.9) | 1.30 | (0.18) | 2627,460 | (7,726,859) | 0.86 | (0.07) |
|  | | ≥ 0.9547 to < 0.9766 | 19 | (40.4) | 1.26 | (0.17) | 417,926 | (1,384,700) | 0.88 | (0.07) |
|  | | ≥ 0.9766 | 13 | (27.7) | 1.26 | (0.14) | 2,057,416 | (6,427,053) | 0.86 | (0.08) |
| P-value | | |  |  | 0.672 | | 0.489 | | 0.652 | |
|  | | |  |  |  |  |  |  |  |  |
| ^1^Stage was missing for 1 patient | | | | | | | | | | |

| **Table B. Concordance correlation coefficient (CCC) and dynamic range for CT features in the test-retest experiment, averages over tumor volume.** | | | |
| --- | --- | --- | --- |
| **Feature** | **Metrics to Test Repeatability** | | **Absolute Percent**  **Difference (\|Test-Retest\|)**  **statistics (µ , σ)** |
|  | **CCC** | **DR** |  |
| Convexity | 0.887667 | 0.924985 | (3.77%, 4.19) |
| Entropy of tumor core | 0.894919 | 0.944633 | (1.68%, 2.04) |
| Entropy of tumor boundary | 0.816676 | 0.927453 | (1.95%,2.69) |
| Entropy Ratio | 0.356657 | 0.914966 | (1.69%,2.58) |

| **Table C. Feature Reproducibility in test-retest analyses using matching center slice on the CT image.** | | |
| --- | --- | --- |
| **Feature** | **CCC** | **DR** |
| Convexity | 0.989043 | 0.971538 |
| Entropy of tumor core | 0.992352 | 0.968886 |
| Entropy of tumor boundary | 0.992078 | 0.968946 |
| Entropy Ratio | 0.997684 | 0.987559 |

**Table D. Clinical parameters and feature scores for Cohort 1 patients.**

| Patient Identifier | TNM Pathology | Vital Status | Survival Time  (months) | | Convexity  Score | Entropy Ratio Score |
| --- | --- | --- | --- | --- | --- | --- |
| Patient_004 | 2B | 1 (ALIVE) | | 44 | 0.895941 | 1.637799599 |
| Patient_006 | 1A | 1 (ALIVE) | | 34 | 0.892942 | 2.051510628 |
| Patient_013 | 1B | 1 (ALIVE) | | 50 | 0.80782 | 1.273299753 |
| Patient_014 | 1A | 1 (ALIVE) | | 25 | 0.940532 | 1.505925886 |
| Patient_019 | 1A | 0 (DEAD) | | 3 | 0.860215 | 1.476915543 |
| Patient_020 | N/A | 0 (DEAD) | | 7 | 0.936766 | 1.450419311 |
| Patient_022 | 2B | 0 (DEAD) | | 15 | 0.852905 | 1.350108967 |
| Patient_029 | N/A | 0 (DEAD) | | 10 | 0.787763 | 1.389544112 |
| Patient_033 | 2A | 0 (DEAD) | | 9 | 0.855026 | 1.145383741 |
| Patient_035 | 3B | 1 (ALIVE) | | 40 | 0.965196 | 1.333453072 |
| Patient_036 | 1B | 0 (DEAD) | | 18 | 0.876594 | 1.421216574 |
| Patient_043 | 3A | 1 (ALIVE) | | 24 | 0.745247 | 1.185617798 |
| Patient_053 | 2B | 0 (DEAD) | | 28 | 0.56621 | 1.539657174 |
| Patient_056 | 4 | 1 (ALIVE) | | 46 | 0.855807 | 0.923998156 |
| Patient_061 | 1A | 1 (ALIVE) | | 31 | 0.820135 | 1.003796846 |
| Patient_064 | 2B | 1 (ALIVE) | | 53 | 0.897708 | 1.293024652 |
| Patient_065 | 3A | 0 (DEAD) | | 12 | 0.875437 | 1.514122013 |
| Patient_066 | 3B | 0 (DEAD) | | 16 | 0.877111 | 1.517057645 |
| Patient_069 | 2B | 0 (DEAD) | | 20 | 0.805064 | 1.892848603 |
| Patient_075 | 1B | 1 (ALIVE) | | 29 | 0.917354 | 1.589426464 |
| Patient_077 | 1A | 1 (ALIVE) | | 48 | 0.927001 | 1.057481677 |
| Patient_078 | 1A | 1 (ALIVE) | | 36 | 0.91025 | 1.081908218 |
| Patient_093 | 3A | 1 (ALIVE) | | 39 | 0.973508 | 1.437448628 |
| Patient_098 | 3A | 0 (DEAD) | | 10 | 0.791397 | 1.233049848 |
| Patient_102 | 2B | 1 (ALIVE) | | 52 | 0.918841 | 1.175298586 |
| Patient_108 | 3A | 0 (DEAD) | | 15 | 0.939503 | 1.494887372 |
| Patient_111 | 2B | 1 (ALIVE) | | 31 | 0.88193 | 1.51369054 |
| Patient_116 | 1A | 1 (ALIVE) | | 24 | 0.666667 | 1.244652643 |
| Patient_117 | N/A | 0 (DEAD) | | 9 | 0.790491 | 1.033623124 |
| Patient_124 | 1A | 1 (ALIVE) | | 39 | 0.897516 | 1.033843648 |
| Patient_126 | 3B | 0 (DEAD) | | 7 | 0.889436 | 1.552041887 |
| Patient_127 | 3A | 0 (DEAD) | | 8 | 0.846932 | 1.434631129 |
| Patient_137 | 3A | 1 (ALIVE) | | 9 | 0.9411 | 1.415481918 |
| Patient_141 | 2B | 1 (ALIVE) | | 6 | 0.864421 | 1.505524832 |
| Patient_143 | 1A | 1 (ALIVE) | | 59 | 0.898634 | 1.401262263 |
| Patient_144 | 2B | 0 (DEAD) | | 10 | 0.958028 | 1.746048258 |
| Patient_146 | 2B | 1 (ALIVE) | | 71 | 0.913239 | 1.32634188 |
| Patient_150 | 1A | 1 (ALIVE) | | 44 | 0.899007 | 1.229961722 |
| Patient_157 | 3A | 0 (DEAD) | | 12 | 0.89019 | 1.406529718 |
| Patient_168 | 3A | 1 (ALIVE) | | 9 | 0.890522 | 2.188790964 |
| Patient_170 | 1A | 0 (DEAD) | | 18 | 0.893387 | 1.40040846 |
| Patient_172 | 2B | 1 (ALIVE) | | 40 | 0.778899 | 1.11975251 |
| Patient_175 | 2A | 0 (DEAD) | | 23 | 0.942142 | 1.523694504 |
| Patient_180 | 1A | 1 (ALIVE) | | 48 | 0.894826 | 1.36175218 |
| Patient_185 | 3A | 1 (ALIVE) | | 32 | 0.889856 | 1.345810989 |
| Patient_191 | 3A | 0 (DEAD) | | 13 | 0.87234 | 1.578902125 |
| Patient_193 | 1B | 0 (DEAD) | | 28 | 0.67382 | 1.26032436 |
| Patient_208 | 2A | 1 (ALIVE) | | 35 | 0.916822 | 1.47792144 |
| Patient_209 | 1B | 1 (ALIVE) | | 27 | 0.885131 | 1.843202606 |
| Patient_210 | 2B | 1 (ALIVE) | | 34 | 0.845553 | 1.613904366 |
| Patient_214 | 2B | 0 (DEAD) | | 17 | 0.889868 | 1.620078459 |
| Patient_232 | 1A | 1 (ALIVE) | | 30 | 0.908654 | 1.712589592 |
| Patient_233 | 1A | 1 (ALIVE) | | 29 | 0.907859 | 1.73328033 |
| Patient_237 | 1A | 1 (ALIVE) | | 42 | 0.89646 | 1.182347065 |
| Patient_239 | 2B | 0 (DEAD) | | 36 | 0.752475 | 1.06587384 |
| Patient_256 | 3B | 1 (ALIVE) | | 54 | 0.732521 | 1.366254758 |
| Patient_259 | 2B | 1 (ALIVE) | | 59 | 0.84766 | 1.137637745 |
| Patient_260 | 1B | 0 (DEAD) | | 17 | 0.900088 | 1.288138825 |
| Patient_266 | 2A | 1 (ALIVE) | | 51 | 0.927613 | 1.745517414 |
| Patient_267 | 1B | 1 (ALIVE) | | 51 | 0.8488 | 1.444262611 |
| Patient_273 | 3 | 1 (ALIVE) | | 49 | 0.895249 | 1.381196087 |
| Patient_274 | 1A | 1 (ALIVE) | | 45 | 0.919811 | 1.041278582 |
